# PReS-FINAL-2084: Prevalence of cervical spine involvement in children with juvenile idiopathic arthritis

**DOI:** 10.1186/1546-0096-11-S2-P96

**Published:** 2013-12-05

**Authors:** N Semolič, N Toplak, J Koder, D Ključevšek, T Avčin

**Affiliations:** 1 Pediatrična Klinika, Ljubljana, Slovenia; 2 Clinical Institute of Radiology, Ljubljana, Slovenia

## Introduction

Cervical spine arthritis is a well-recognized complication of juvenile idiopathic arthritis (JIA). It is usually present in patients with more severe disease.

## Objectives

To evaluate the prevalence of symptomatic cervical spine involvement and their association with MRI findings in children with JIA.

## Methods

The study was a retrospective study of all patients with JIA followed at University Children's Hospital Ljubljana from September 2010 to January 2013. We reviewed the charts of 206 patients with JIA diagnosed according to the ILAR criteria. Clinical, laboratory data and MRI of cervical spine where available were collected and reviewed.

## Results

206 patients, 143 girls (69%) and 63 boys (31%) with JIA were followed at University Children's Hospital Ljubljana from September 2010 to January 2013. Twenty-two out of 206 (11%) patients had pain in the neck and/or limited range of motion of cervical spine. Twelve out of 206 (6%) patients had persistent cervical spine symptoms, MRI of the cervical spine with application of paramagnetic contrast was performed in those. The results revealed inflammation of atlantoaxial (AA) joint without subluxation in 6/206 (3%) patients. Among 6 patients with AA joint inflammation were 5 girls and one boy. Three of them had RF negative polyarthritis, two persistent oligoarthritis and one girl had juvenile psoriatic arthritis. Five out of six (83%) patients were ANA positive and none of them had positive HLA B-27 antigen. In four out of six patients (66%) with AA joint inflammation, the neck pain was an initial manifestation of JIA. Five out of six patients (83%) were treated with anti-TNF alpha agents and demonstrated good clinical recovery.

## Conclusion

The prevalence of AA joint inflammation in our cohort of patients with JIA was 3%. Cervical spine involvement could present as an initial manifestation of JIA and in 83% of our cases demonstrated good clinical response on treatment with anti-TNF alpha agents.

## Disclosure of interest

None declared.

